# Short Echo‐Time Spiral MRSI Versus Single‐Voxel Spectroscopy in the Human Brain at 3 Tesla With Semi‐LASER Localization

**DOI:** 10.1002/mrm.70463

**Published:** 2026-06-05

**Authors:** Young Woo Park, Isaac M. Adanyeguh, Pierre‐Gilles Henry, Dinesh K. Deelchand

**Affiliations:** ^1^ Center for Magnetic Resonance Research, Department of Radiology University of Minnesota Minneapolis Minnesota USA; ^2^ Henry M. Jackson Foundation for the Advancement of Military Medicine, Inc Bethesda Maryland USA; ^3^ Department of Radiology Uniformed Services University of the Health Sciences Bethesda Maryland USA

## Abstract

**Purpose:**

Magnetic resonance spectroscopy techniques are widely used to non‐invasively study brain metabolism. Despite advances in magnetic resonance spectroscopic imaging (MRSI), there is a notable absence of research on comparing fast non‐Cartesian MRSI with single‐voxel spectroscopy (SVS), limiting our understanding of its performance and applicability. In this study, we compared the spectral quality and metabolite concentrations obtained using short‐T_E_ 2D spiral MRSI and SVS in the same region in the human brain at 3T.

**Methods:**

Five healthy subjects were scanned at 3T. 2D spiral MRSI data were acquired in a transverse slice through the posterior cingulate cortex (PCC), while the SVS volume was placed within the PCC region. Both techniques employed the standardized semi‐LASER sequence for localization. All data were processed in Matlab and fitted with LCModel.

**Results:**

Visual inspection suggested comparable overall spectral quality between the two acquisitions. Quantitatively, however, the PCC voxel measured with MRSI exhibited lower signal‐to‐noise ratio (SNR) compared to SVS at identical scan times, when the SVS voxel matched the effective MRSI voxel. Consistent with lower SNR, metabolite quantification showed higher Cramer‐Rao lower bounds with MRSI. In addition, concentrations of glutamate and glutamate plus glutamine were lower with MRSI.

**Conclusion:**

Our findings demonstrate that the quality of semi‐LASER localized short‐TE spiral MRSI spectra is very comparable to that of semi‐LASER localized SVS spectra. Small metabolic‐specific concentration differences may be due to different WM/GM tissue weighting within the voxel (slice selection profile in SVS vs. point‐spread function in MRSI) and different SNR between the two techniques.

## 1 Introduction

Proton magnetic resonance spectroscopy (^1^H MRS) is a non‐invasive technique that enables quantification of various brain metabolites in vivo using clinical MR scanners. MRS data can be acquired using either single‐voxel spectroscopy (SVS) or with multi‐voxel magnetic resonance spectroscopic imaging (MRSI) [1]. SVS is commonly used due to high spectral quality, benefiting from precise localization, optimized B_0_ shimming and effective water suppression such as VAPOR [2]. The high spectral quality of short‐T_E_ SVS enables reliable quantification of metabolites with complex spectral patterns, such as glutamate and glucose, for better understanding of the metabolic changes and responses in the brain. In general, SVS offers superior quantification precision and accuracy relative to MRSI [3, 4].

Despite these advantages, SVS is limited to a single volume‐of‐interest (VOI) at a time, restricting spatial coverage and requiring precise VOI placements. In contrast, MRSI data are acquired simultaneously from multiple VOIs across one or multiple slices, providing more comprehensive spatial information [5]. This broader coverage makes MRSI a promising tool for clinical assessment of various neurological diseases such as tumors, metabolic diseases, and neurodegeneration. Nevertheless, despite its potential, conventional MRSI has historically been largely confined to research settings due to prohibitively long acquisition times.

However, recent advances in fast MRSI acquisition techniques [6, 7] have significantly reduced scan durations, improving feasibility for clinical use. Among these, spiral MRSI stands out for its high sampling efficiency in *k*‐space and compatibility with various volume excitation strategies. Yet, few studies have compared SVS and MRSI techniques in the human brain, and most of them used the conventional Cartesian MRSI with long scan time [3, 4]. Recently, a concentric ring trajectory MRSI approach compared to SVS was reported, demonstrating reproducible metabolite measurements and bridging the gap between single‐voxel and fast multiregional acquisitions [8]. Despite these developments, it remains unclear how fast spiral MRSI compares to SVS in spectral quality and metabolite quantification, particularly at clinically relevant field strengths following recent technological advances in the field [7, 9].

In this study, we present a direct comparison between SVS and pre‐localized spiral MRSI results in the human brain at 3 Tesla, using semi‐LASER for voxel localization. We assess the spectral quality and metabolite concentrations obtained with short‐T_E_ 2D spiral MRSI relative to SVS with matched acquisition parameters. Finally, we discuss the respective strengths and limitations of each method, and the broader implications for using spiral MRSI in clinical and research applications.

## 2 Methods

### 2.1 Hardware and Participants

Five healthy participants (mean age 40 ± 25, 2 male) were scanned for this study. The study protocol was approved by the Institutional Review Board (IRB) at the University of Minnesota prior to participant recruitment, and all participants provided written informed consent.

All data were acquired using a Siemens 3T Prisma Fit scanner (Syngo VE11C; Erlangen, Germany). The standard body coil was used for RF pulse transmission, and a 20‐channel head‐and‐neck matrix coil was used for signal reception.

### 2.2 Data Acquisition

**FIGURE 1:**
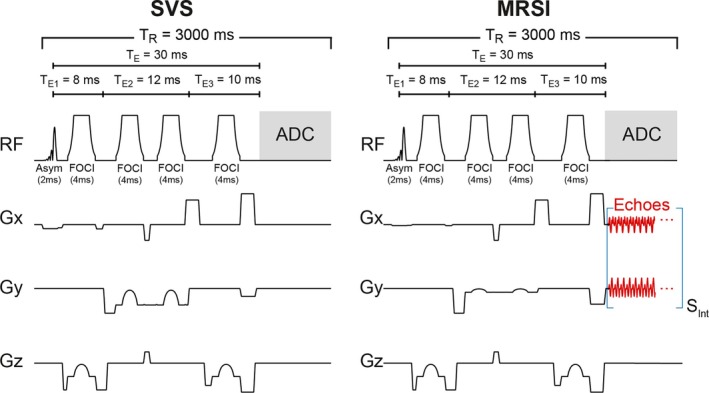
Pulse sequence diagrams comparing the semi‐LASER pulse sequences for SVS and spiral MRSI. Identical RF pulse shapes, durations, and T_E_ were used for both techniques. The MRSI diagram shows the spiral‐out gradients, including the rewinder gradients, for the first spatial interleaf (S_Int_) out of five used in this study. For SVS, the VOI was 18 × 18 × 20 mm^3^ whereas the MRSI VOI was 80 × 80 × 20 mm^3^.

Figure 1 shows the pulse sequence diagrams for both SVS and MRSI acquisitions. All MRS data (SVS and MRSI) were localized using semi‐LASER [2, 10] with T_R_ of 3 s and T_E_ of 30 ms (T_E1_/T_E2_/T_E3_ = 8/12/10 ms). An asymmetric amplitude‐modulated RF pulse of 2 ms was used for excitation (bandwidth = 3.39 kHz) and FOCI adiabatic pulses [11] of 4 ms (bandwidth = 12.5 kHz) were used for refocusing. FOCI refocusing pulses were chosen because they provide sharper VOI profiles (figure 3 in Deelchand et al. [12]) compared with other adiabatic pulses such as GOIA, and our Prisma scanner provides sufficient B_1_^+^ to meet the adiabaticity condition. Metabolite cycling (MC) technique was employed to suppress the water signal, and outer volume suppression modules were applied to minimize lipid contamination [13] and the carrier frequency was set to at 2.65 ppm.

**FIGURE 2:**
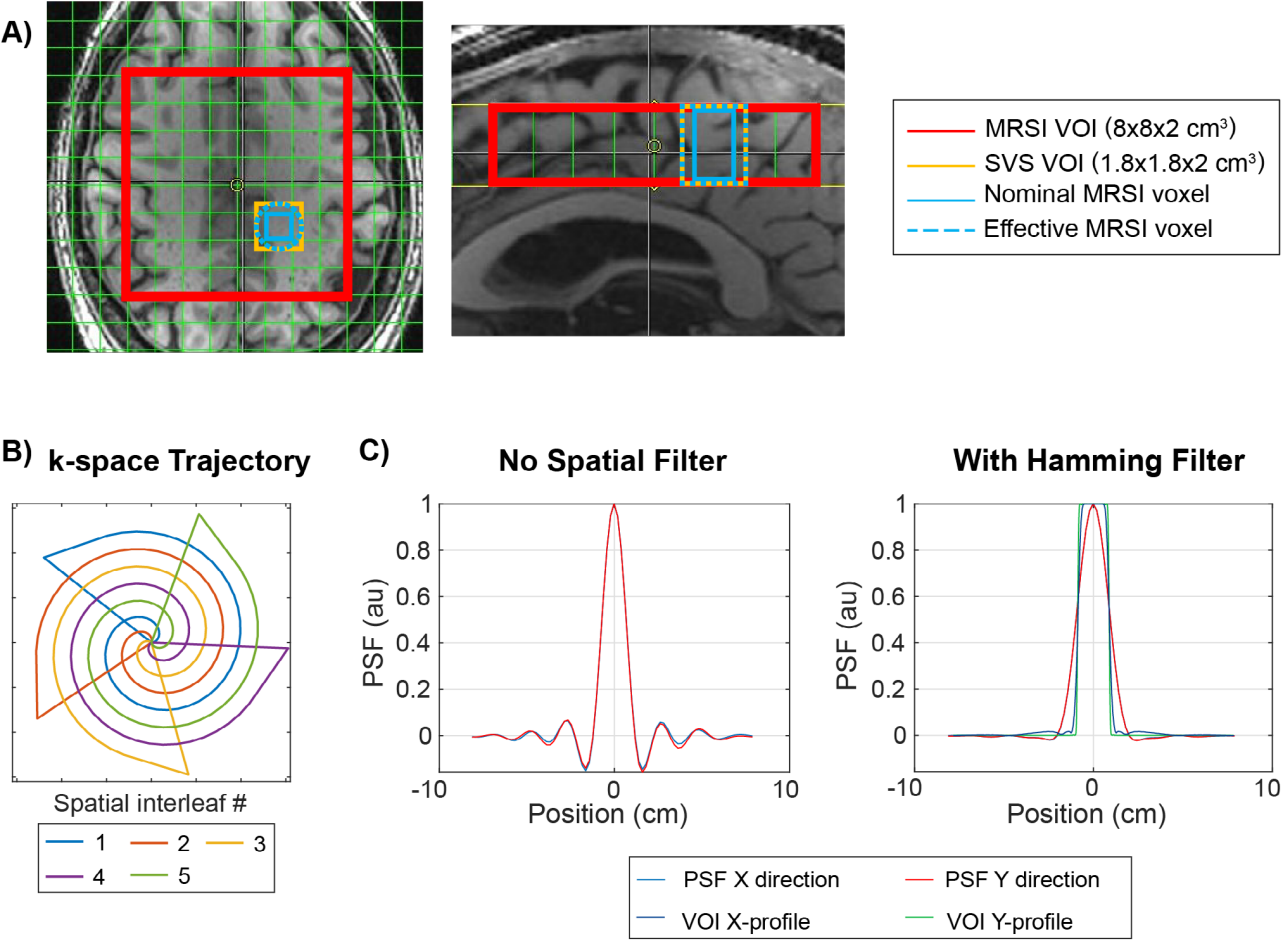
(A) Location of MRSI VOI (red box) with its associated grids (green lines) and the SVS VOI (orange box) on anatomical images. The SVS VOI was positioned in the PCC region (which contained a mixture of white matter) and gray matter and the corresponding MRSI voxels at this position (nominal voxel: Solid cyan box; effective voxel: Dashed cyan circle). (B) *k*‐space trajectory of the spiral‐out MRSI acquisition, showing all five spatial interleaves including the rewinder at the end. (C) The point‐spread function (PSF) distributions from the spiral MRSI acquisition without (left) and with (right) spatial filtering. PSF profiles along the X and Y directions are shown, along with the VOI profiles obtained from Bloch simulations.

MRSI data were acquired from a semi‐LASER localized axial slice (80 × 80 × 20 mm^3^) positioned above the corpus callosum, encompassing the posterior cingulate cortex (PCC), as shown in Figure 2A. The MRSI slice dimension was selected to exclude the scalp and thereby minimize lipid contamination in the MRSI data. Spiral‐out readout gradients were used to simultaneously encode two spatial and one frequency dimension (FOV = 160 × 160 mm^2^, matrix = 16 × 16, nominal resolution = 10 × 10 × 20 mm^3^, slew rate = 150 T/m/s, receiver bandwidth = 100 kHz). To achieve a spectral bandwidth (SBW) of 1.14 kHz, spiral readout with five spatial interleaves was used (Figure 2B). Each interleaf was acquired 20 times, with MC pulses applied in an alternating upfield/downfield pattern every five shots, resulting in 20 excitations per interleaf and 20 FIDs after reconstruction (10 with upfield and 10 with downfield MC pulses). During each interleaf, the spiral readout gradient was repeated 400 times, providing 400 complex points per voxel for each FID. The total acquisition time was 5 min (20 repetitions × 5 interleaves × 3 s TR).

For the SVS acquisition (SBW = 6 kHz, 2048 complex points), a VOI of 18 × 18 × 20 mm^3^ (Figure 2A) was selected to match the effective MRSI voxel size, approximately 18 mm at the point‐spread function (PSF) FWHM following spatial filtering (Figure 2C). A total of 100 metabolite transients were acquired with a total scan time of 5 min (100 repetitions × 3 s TR). This VOI was positioned at the same location as the PCC VOI of MRSI, which contained a mixture of white matter and gray matter.

B_0_ shimming was performed using FAST(EST)MAP [14] with four iterations on the larger MRSI VOI (80 × 80 × 20 mm^3^) and the same shim settings were subsequently applied for the SVS acquisition (18 × 18 × 20 mm^3^). Metabolite and water reference scans data were collected for both SVS and MRSI acquisitions. Additionally, T_1_‐weighted MPRAGE images were acquired for localization of the MRSI slice and SVS voxels, and for estimating tissue and cerebrospinal fluid fractions for metabolite quantification. The reference voltage for semi‐LASER was calibrated as previously described [15].

### 2.3 Data Processing

Raw MRSI data (uncombined, complex *k*‐space signals from each receive coil) were reconstructed in MATLAB, yielding a 16 × 16 grid of spectra corresponding to the MRSI voxels. The interleaved spiral *k*‐space data were converted to Cartesian coordinates using the non‐uniform fast Fourier transform, followed by density compensation, 2D FFT for spatial encoding and 1D FFT along the spectral dimension. The effective volume of the MRSI voxel was estimated using the PSF. A single‐voxel point source was simulated using the spiral gradient waveforms from the in vivo acquisition and reconstructed with the same pipeline, including the Hamming filter. From the PSF, the FWHM was measured as 18 mm in‐plane after Hamming filtering, Here we assumed a cuboid VOI where the effective voxel volume is 18 × 18 × 20 mm^3^ = 6.48 mL. In reality the in‐plane VOI is circular (reflecting the PSF shape), and the volume is Π (18/2)^2^ × 20 mm^3^ = 5.09 mL.

All spectroscopic data (SVS and reconstructed MRSI) were processed using MRspa [16] following identical processing steps as recently described for MC data [17]. For SVS, spectra were saved with 20 and 100 transients; 20 to match the number of transients in MRSI and 100 to match the acquisition time of MRSI.

All summed spectra were quantified using LCModel (v.6.3‐1R) [18]. The basis set, which both SVS and MRSI analysis shared, included both an experimentally acquired multi‐subject averaged macromolecule (MM) basis spectrum as well as density matrix‐simulated basis spectra of aspartate (Asp), gamma‐aminobutyric acid (GABA), glucose (Glc), glutamate (Glu), glutamine (Gln), glutathione (GSH), *myo*‐inositol (Ins), phosphoethanolamine (PE), phosphocholine (PCho), glycerylphosphorylcholine (GPC), creatine (Cr), phosphocreatine (PCr), *N*‐acetylaspartate (NAA), *N*‐acetyl‐aspartylglutamate (NAAG), lactate (Lac), and taurine (Tau). Water scaling was used for metabolite quantification, incorporating the PSF‐corrected [19] tissue fractions (white matter, gray matter, and cerebrospinal fluid) within the PCC for each subject and assuming a water T_2_ relaxation time of 120 ms [20]. Signal loss arising from metabolite T_2_ relaxation was neglected. Metabolite ratios relative to tCr were also reported.

The full‐width‐half‐maximum values (linewidth) of the water reference spectra were used as a quality control metric during the acquisition. For MRSI, the water linewidths of both the entire MRSI slice and the separate PCC volume are reported.

Signal‐to‐noise ratio (SNR) was measured by taking the amplitude of NAA singlet at 2 ppm over the root mean square error of noise measured from a signal‐free region of the spectrum (SNR_NAA_). In addition, a normalized SNR of NAA per unit volume and unit time (SNR_norm_) was computed to compare the sensitivity between the SVS and MRSI [21]. The unit volume was the VOI measured in cubic centimeters and the unit time was the total acquisition time in minutes. Since a Hamming spatial filtering is applied during the reconstruction of the MRSI data, the reported SNR values for the MRSI data reflect the effective voxel size after filtering. Differences in SNR, linewidth, and metabolites concentration between the two techniques were assessed using a paired two‐tailed *t*‐test.

For SNR analysis, multiple comparisons between SVS (NT = 100) and SVS (NT = 20) versus MRSI were controlled using a Bonferroni correction (*α* = 0.05/2 = 0.025). For concentration and CRLB analysis, multiple comparisons across seven metabolites were controlled separately for each method comparison (SVS NT = 100 vs. MRSI and SVS NT = 20 vs. MRSI) using Bonferroni correction (*α* = 0.05/7 = 0.0071). For relative concentration analysis, multiple comparisons across six metabolites were controlled separately for each method comparison using Bonferroni correction (*α* = 0.05/6 = 0.0083).

## 3 Results

### 3.1 MRS Data Quality Comparison

**FIGURE 3:**
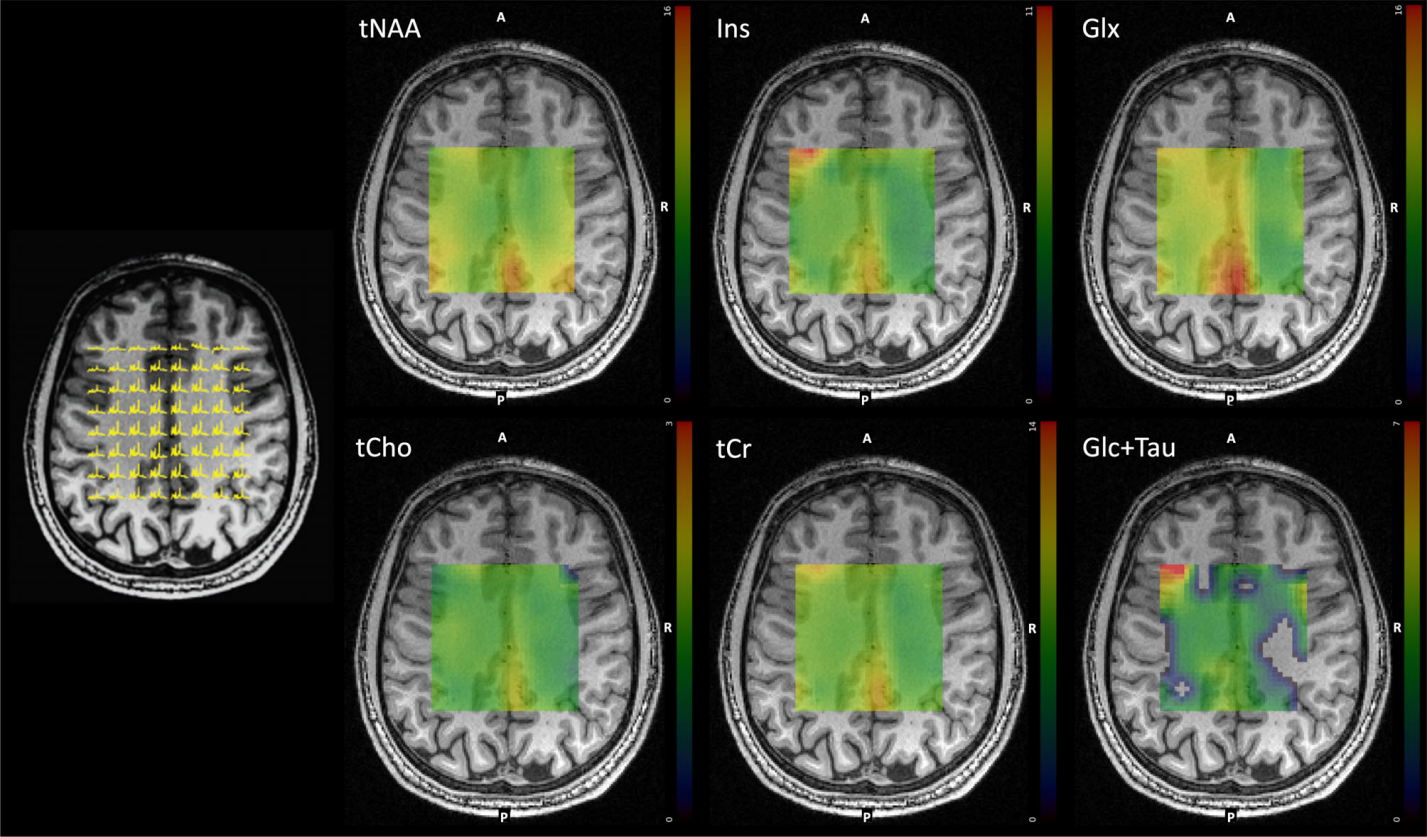
Metabolite maps (mM) generated by MRSI (4× interpolated) in one subject (S4) show differing levels of concentration in different tissue types. The 2D MRSI data show high‐quality spectra (left).

Figure 3 presents an example of MRSI dataset from a single subject (total scan time: 5 min), showing high spectral quality with minimal lipid contamination. High‐quality short‐TE semi‐LASER spiral MRSI data were acquired from all participants. The average water linewidth (full‐width–half‐maximum), measured after FAST(EST)MAP, was 10.3 ± 1.1 Hz across the large MRSI VOI. For SVS, the water linewidth was 6.8 ± 0.8 Hz.

**FIGURE 4:**
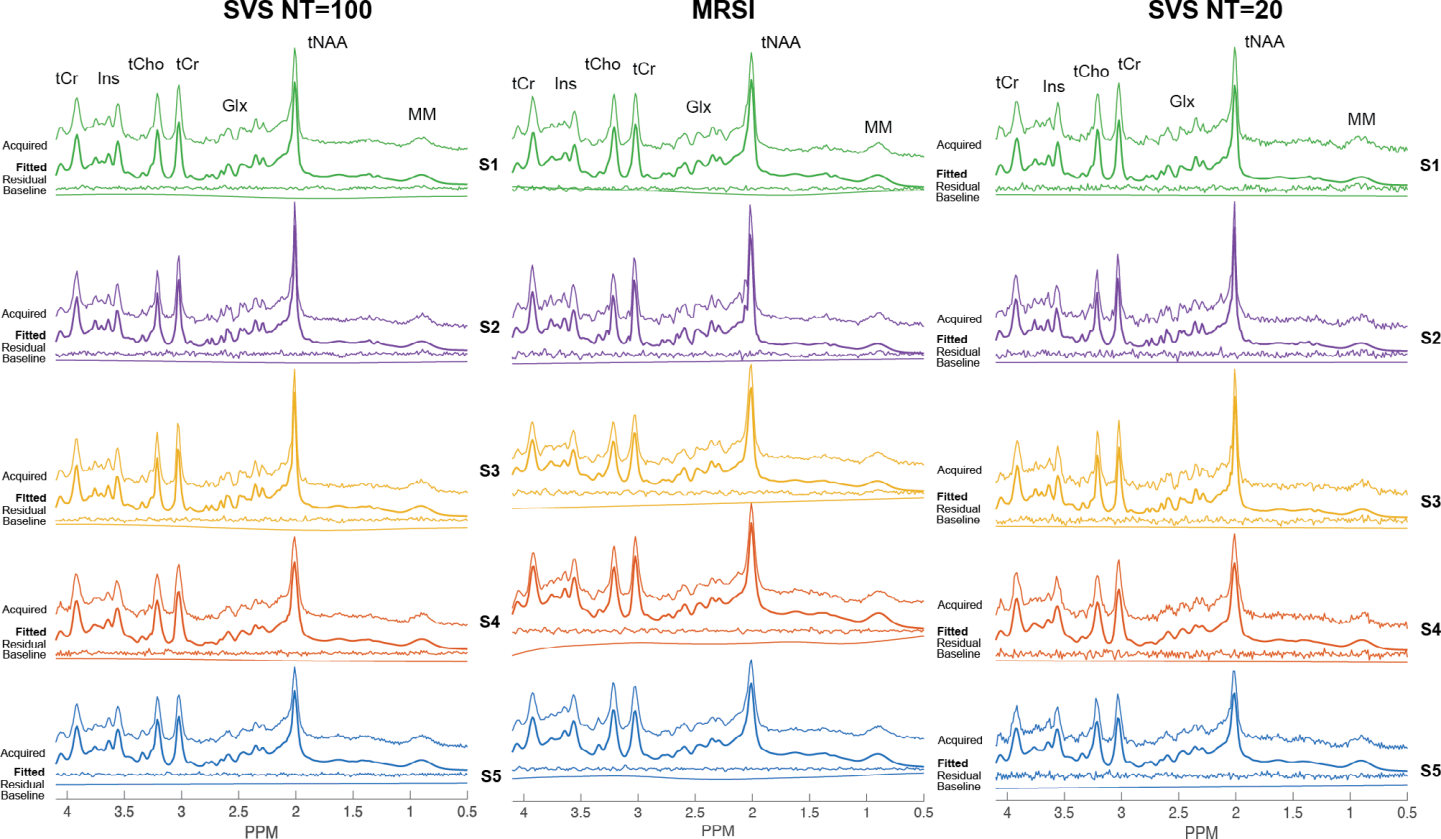
Comparison of SVS with 100 transients (NT = 100, left), MRSI (center) and SVS with 20 transients (NT = 20, right) semi‐LASER spectra (T_R_/T_E_ = 3000/30 ms) from the PCC region for each participant (S1 to S5). For each participant, plots show (from top to bottom) the acquired spectrum, the LCModel fitted spectrum, the residuals and the spline baseline. Similar spectral patterns were observed between the three different groups, with minor differences in the tCr to tCho singlet ratio between the SVS methods and MRSI. For display purposes, 2X zero padding and a Gaussian weighting of 0.15 s was applied.

A comparison between SVS data and the corresponding MRSI data in the PCC revealed similar spectral quality and patterns across subjects (Figure 4). Although identical B_0_ shimming settings were used in both acquisitions, SVS demonstrated a slightly narrower water linewidth in the PCC voxel than in MRSI in the corresponding VOI (7.7 ± 1.0 Hz), although the difference was not significant (*p* = 0.224 for paired two‐tailed *t*‐test). Despite similar spectral patterns, minor differences in the tCr to tCho singlet ratio were observed in a few subjects. While the segmented tissue fractions within the nominal PCC VOI did not differ significantly between SVS and MRSI (Table S1), the broader PSF of the MRSI acquisition results in a different spatial weighting compared to the sharper SVS VOI profile (Figure 2C). Consequently, the MRSI voxel appears to contain slightly different GM and WM tissue composition compared to the SVS voxel on a per‐subject basis.

The SNR metrics are summarized in Table S2. The measured SNR_NAA_ was significantly higher for SVS with 100 transients compared to the overlapping voxel from the MRSI slice (118.5 ± 16.3 vs. 67.0 ± 7.1, *p* = 0.0002). A similar difference (ratio∼1.77) was observed when comparing the normalized SNR per unit volume per time, as the acquisition time was matched between the two acquisitions. No statistical difference in SNR_NAA_ was also observed between SVS data with 20 transients and the MRSI data after Bonferroni correction.

### 3.2 Metabolite Concentration Estimates of SVS and MRSI

LCModel analysis yielded reliable quantification, defined by the mean Cramér‐Rao lower bounds (CRLB) under 20% for 17 neurochemicals in SVS and 11 in MRSI. Cross‐correlation analysis of LCModel fits revealed strong negative correlations between certain metabolite pairs. These pairs were reported as summed values: Cr and PCr (−0.91 for SVS and −0.82 for MRSI) summed as total creatine (tCr); PCho and GPC (−0.83 for SVS and −0.83 for MRSI) summed as total choline (tCho); NAA and NAAG (−0.54 for SVS and −0.64 for MRSI) summed as total NAA (tNAA); Glc and Tau (−0.58 for SVS and −0.63 for MRSI) summed as Glc + Tau. Additionally, the combined spectrum of Glu and Gln was reported as Glx (−0.40 for SVS and −0.50 for MRSI). In total, seven different metabolites were reliably quantified for both SVS and MRSI and were used for comparison: Glu, Ins, tCho, tCr, tNAA, Glx, and Glc + Tau.

**FIGURE 5:**
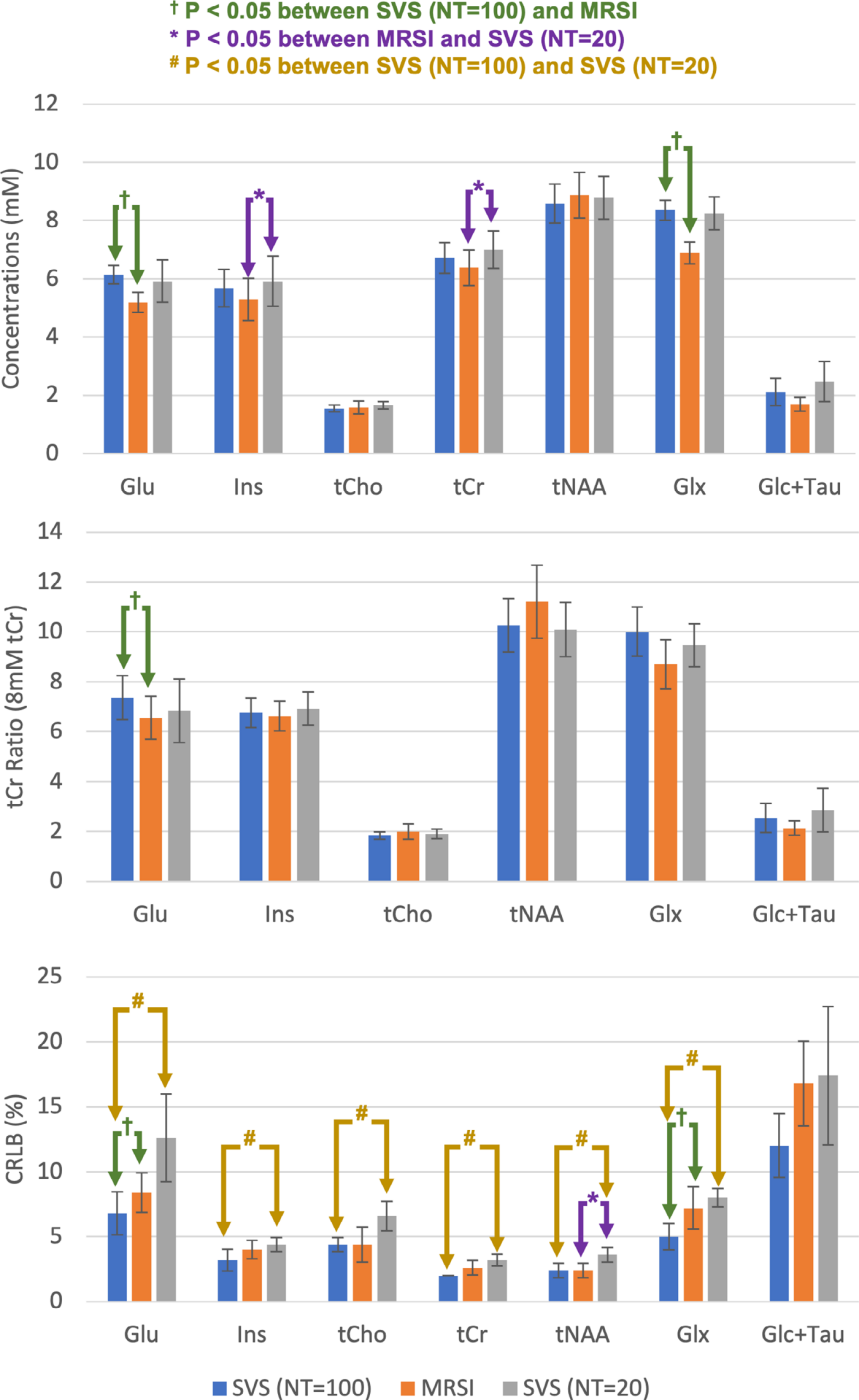
Mean metabolite concentrations (tissue fractions and water tissue T_2_ corrected), concentrations relative to tCr and CRLBs. Data are shown for SVS with full 100 transients (blue), MRSI (orange) and the SVS with only 20 transients (gray). Error bars represent standard deviations. † indicates a significant difference between full SVS (NT = 100) and MRSI, * represents a significant difference between SVS with 20 transients (NT = 20) and MRSI, and # shows a significant difference between SVS (NT = 100) and (NT = 20). All comparisons (paired, two‐tailed *t*‐test) were corrected using Bonferroni correction.

Signals from major metabolites such as tNAA, tCr, tCho, and Ins, as well as other lower concentration metabolites, were clearly visible in all datasets (Figure 4). Mean concentrations of Glu and Glx were significantly lower in spiral MRSI compared to SVS with 100 transients (Figure 5). LCModel analysis showed slightly reduced precision in concentration estimates, with CRLB values significantly higher for Glu and Glx. Decreasing trends in Ins and tCr, as well as an increasing trend in tNAA, were observed with MRSI, although these differences were not statistically significant.

When comparing MRSI with SVS using 20 transients, Ins, and tCr showed statistically significant differences in concentration. In contrast, CRLB values were significantly different for tNAA only. Comparison across different numbers of transients in SVS showed no significant differences in concentrations, while the CRLB values were significantly different for all metabolites except Glc + Tau. A strong positive correlation was observed between SVS (100 transients) and MRSI concentrations for Ins, tCho, tNAA, and tCr (Pearson *r* = 0.89, 0.97, 0.95, and 0.93, respectively).

When metabolite ratios relative to tCr were compared, only Glu showed statistically significant differences between SVS and MRSI (Figure 5).

Overall, differences in concentrations between MRSI and SVS were relatively small (< 25%). The largest difference was observed for Glu + Tau (25% lower with MRSI than with SVS) followed by Glx (21%) and all other differences were in the 5%–15% range.

## 4 Discussion

In this study, we compared short‐T_E_ semi‐LASER MRS data acquired using SVS and spiral MRSI in the same five participants, using identical acquisition parameters, including semi‐LASER localization and B_0_ shim settings. While spectral quality appeared to be visually comparable, we found small differences in the concentration estimates of select metabolites across the two acquisition approaches.

### 4.1 Similarities and Differences

With identical scan times, SVS demonstrated superior metabolite quantification performance compared to spiral MRSI, as evidenced by the number of reliably quantified metabolites (group mean CRLB < 20%) and lower mean CRLB values (Figure 5). This is due in part to SVS having higher SNR_NAA_ than spiral MRSI when compared on a per‐voxel basis at the same effective volume, despite the latter's advantage in larger spatial coverage. Although spatial filtering was applied during the reconstruction of the MRSI data and the VOI of the SVS was matched to yield the same effective voxel size, resulting in comparable spectral patterns, differences in the measured and normalized SNR were still noticed. One likely explanation is that continuously varying gradients in spiral MRSI broaden the PSF along the k‐t trajectory (Figure 2C), reducing per‐voxel peak height and SNR efficiency [22]. Note that while the effective bandwidth was different for SVS (6 kHz) and MRSI (1.14 kHz), this should not introduce a difference in SNR since the fid duration was comparable between acquisitions (SVS: 342 ms; MRSI: 352 ms) [23]. Lastly, spatial crosstalk arising from the broader PSF of spiral MRSI may lead to discrepancies due to contributions from neighboring voxels, which is minimized in SVS with a sharper VOI profile.

Beyond these technical factors, tissue composition effect may also contribute to the observed differences. Due to the PSF of the MRSI scan, a slightly larger WM contribution is expected within the PCC voxel. Previous studies [24, 25, 26, 27] have shown that concentrations of tNAA, tCr, Ins, and Glu are higher in GM than in WM, whereas tCho exhibits the opposite trend. These findings are consistent with the current results, where Glu and Glx were significantly lower in MRSI. The observed trends for other metabolites, such as Ins, tCho, and tNAA, are also in agreement with known GM and WM differences [24, 25]. Notably, the GM and WM contrast difference is typically greater for Glu, which may explain why Glu and Glx showed statistically significant differences compared to others.

When matching the number of transients between MRSI and SVS, the observed differences in concentrations remained consistent with the results obtained using equal total scan time. However, CRLB values were higher in SVS, consistent with lower SNR_NAA_ compared to MRSI (Table S2). This indicates that despite equal signal averaging, differences in spatial encoding and SNR continue to influence spectral quality and quantification precision [22].

Although the B_0_ shim was not optimal for the SVS acquisition, the resulting water linewidth in this study was comparable to that reported in other studies where first and second order shim terms were optimized in the PCC [12, 28]. In addition, no significant difference in linewidth was observed between the SVS and MRSI data in the PCC VOI, indicating that B_0_ shimming is unlikely to explain the differences in metabolite concentrations observed between acquisitions.

### 4.2 Novelty Within Context of Prior SVS Versus MRSI Comparisons

Recent advances in MRSI have focused on improving its feasibility, including faster acquisition techniques such as use of spatial‐spectral encoding techniques [9, 29, 30], and the integration of prospective motion tracking [31] to enable robust, highly‐accelerated acquisition of spatial and spectral information. This allowed generation of high‐resolution (voxel dimension of 2 to 3 mm per side) metabolite 2D and 3D maps [32, 33, 34] within scan time that is viable for a clinical study.

However, efforts to cross‐validate the MRSI‐derived metabolite levels against established SVS methods have been limited. As noted earlier, several prior studies primarily compared SVS with conventional Cartesian MRSI techniques [3, 4]. Early investigations reported similar metabolite ratios in the hippocampus between SVS and conventional MRSI acquisitions at long T_E_ [35, 36]. McNab and Bartha [3] used LASER for localization at a long T_E_ of 46 ms, carefully matching the SVS VOI to the corresponding effective MRSI voxel. They found no differences in SNR or linewidth between the acquisitions, and major metabolites were largely similar, with the exception of Glu, which was statistically higher in MRSI. However, these results were reported without correction for tissue composition within the voxels. Another study, by Zhang et al. [4], compared SVS using PRESS (T_R_/T_E_ = 2000/30 ms) with whole brain 3D echo‐planar spectroscopic imaging (T_R_/T_E_ = 1550/17.6 ms). Good reproducibility (< 10%) was observed for the major metabolites (NAA, Cr, Cho, Ins) across techniques, despite differences in localization methods, T_E_ and T_R_. Unfortunately, the authors did not perform any formal quantitative comparison of metabolite concentrations between SVS and MRSI. More recently, Eftekhari et al. [8] compared semi‐LASER SVS with 3D concentric ring trajectory FID‐MRSI at both 3T and 7T. Once again, reproducibility was high for several metabolites across field strength. Yet, consistent with the earlier study, no direct quantitative assessment of metabolite concentrations (or ratios) between SVS and MRSI was conducted, leaving unresolved the question of potential systematic bias between voxel‐based and whole‐brain mapping approaches. In contrast, the present study directly compares SVS with spiral MRSI, representing a meaningful step toward validating and refining accelerated spatial‐spectral MRSI approaches. This work may ultimately facilitate broader adoption of MRSI in both research and clinical settings.

### 4.3 Limitations and Future Directions

We acknowledge several limitations in the current study. First, although we carefully matched acquisition parameters (acquisition time, effective VOI size, number of transients, B_0_) between MRSI and SVS, the spiral MRSI still incurs a modest SNR penalty per voxel, most likely due to residual PSF effects and reconstruction‐related noise, even though it enables acquisition of spatial metabolite maps.

In addition, our comparison focused exclusively on the PCC voxel, which does not account for potential regional variability in spectral quality and quantification. Future studies should extend the analysis to multiple brain regions to determine whether similar trends are observed across different anatomical areas.

Finally, even though both SVS and MRSI data were fitted using the same basis set, some differences in concentrations may arise due to differences in the number of spectral data points used in the fitting range, as well as baseline modeling and noise variance estimation.

## 5 Conclusion

The current study demonstrates that the quality of semi‐LASER localized short‐T_E_ spiral MRSI spectra is very comparable to semi‐LASER localized SVS spectra acquired within the same acquisition time (∼5 min) with SVS having a slight edge in SNR. The small differences in metabolite concentrations observed between the two MRS techniques are likely attributable to differences in PSF distribution and SNR between spiral MRSI and SVS acquisitions. Although concentrations and error estimates were slightly different with MRSI for Glu and Glx, the technique should still be useful for comparing different cohorts or disease states, since the relative differences are preserved.

## Funding

This work was supported by the Office of The Director, National Institutes of Health (OD), (1S10OD017974) and the National Institute of Biomedical Imaging and Bioengineering (P41 EB027061, R01 EB030000).

## Disclosure

The content is solely the responsibility of the authors and does not necessarily represent the official views, opinions or policies of the National Institutes of Health, the Henry M. Jackson Foundation for the Advancement of Military Medicine Inc., the Uniformed Services University of the Health Sciences (USUHS), the Department of War (DoW), or the Departments of the Army, Navy, or Air Force. Mention of trade names, commercial products, or organizations does not imply endorsement by the U.S. Government.

## Conflicts of Interest

The authors declare no conflicts of interest.

## Supporting information


**Table S1:** Mean tissue fractions and *p*‐values from *t*‐tests (paired, two‐tailed) for SVS and MRSI in gray (GM), white matters (WM) and cerebrospinal fluid (CSF) are shown.
**Table S2:** SNR metrics (mean, *N* = 5) for SVS and MRSI with effective volume. SNR_NAA_: the measured SNR of NAA, SNR_norm_: SNR of NAA normalized per unit volume and per unit time. † represents significant differences between SVS (NT = 100) and MRSI, and * indicates significant differences between SVS (NT = 20) and MRSI. No significant differences were found between SVS (NT = 100) and (NT = 20). All comparisons (paired, 2‐tailed t‐test) were corrected using Bonferroni correction.

## Data Availability

The data that support the findings of this study are available on request from the corresponding author. The data are not publicly available due to privacy or ethical restrictions.
