# Supplementary material for: Short Echo‐Time Spiral MRSI Versus Single‐Voxel Spectroscopy in the Human Brain at 3 Tesla With Semi‐LASER Localization
Source: Magn Reson Med. 2026 Jun 5;96(4):1524–33. doi: 10.1002/mrm.70463 (PMC13419330; doi:10.1002/mrm.70463)
Supplement: Supplementary file 1 — Table S1: Mean tissue fractions and p‐values from t‐tests (paired, two‐tailed) for SVS and MRSI in gray (GM), white matters (WM) and cerebrospinal fluid (CSF) are shown. Table S2: SNR metrics (mean, N = 5) for SVS and MRSI with effective volume. SNRNAA: the measured SNR of NAA, SNRnorm: SNR of NAA normalized per unit volume and per unit time. † represents significant differences between SVS (NT = 100) and MRSI, and * indicates significant differences between SVS (NT = 20) and MRSI. No significant differences were found between SVS (NT = 100) and (NT = 20). All comparisons (paired, 2‐tailed t‐test) were corrected using Bonferroni correction. [file MRM-96-1524-s001.docx]

## Supplementary Table

| **(%)** | **GM** | **WM** | **CSF** |
| --- | --- | --- | --- |
| **SVS** | 27.1 ± 5.9 | 71.4 ± 6.2 | 1.5 ± 1.5 |
| **MRSI** | 30.2 ± 12.2 | 67.6 ± 12.8 | 2.2 ± 1.3 |
| **p-value** | 0.48 | 0.45 | 0.33 |

**Table S1**: Mean tissue fractions and p-values from t-tests (paired, 2-tailed) for SVS and MRSI in gray (GM), white matters (WM) and cerebrospinal fluid (CSF) are shown.

| **Methods** | **SNR_NAA_** | **SNR_norm_** |
| --- | --- | --- |
| **SVS (NT=100)** | 118.5 ± 16.3 | 8.2 ± 1.1 |
| **SVS (NT=20)** | 53.5 ± 9.6 | 10.3 ± 1.1* |
| **MRSI** | 67.0 ± 7.1† | 4.6 ± 0.5† |

**Table S2**: SNR metrics (mean, N=5) for SVS and MRSI with effective volume. SNR_NAA_: the measured SNR of NAA, SNR_norm_: SNR of NAA normalized per unit volume and per unit time. † represents significant differences between SVS (NT=100) and MRSI, and * indicates significant differences between SVS (NT=20) and MRSI. No significant differences were found between SVS (NT=100) and (NT=20). All comparisons (paired, 2-tailed t-test) were corrected using Bonferroni correction.
